# Artificial Intelligence-Oriented Heart Surgery: A Complex Bioethical Concept

**DOI:** 10.7759/cureus.41911

**Published:** 2023-07-15

**Authors:** Konstantinos C Christodoulou, Gregory Tsoucalas

**Affiliations:** 1 Department of Cardiac Surgery, University Hospital of Alexandroupolis, Democritus University of Thrace, Alexandroupolis, GRC; 2 Department of History of Medicine and Medical Deontology, School of Medicine, University of Crete, Heraklion, GRC

**Keywords:** ethical challenge, cardiac surgery procedures, machine learning, robotic surgeon, ai & robotics in healthcare

## Abstract

Artificial intelligence (AI) has come to the frontline, paving the way toward a future of operational efficiency. Following the current, cardiac surgery has evolved as well. We live in a world where AI has brought immense progress in almost every aspect of the field, but still, the question remains; will there be a time when robots will replace cardiac surgeons? The currently used operating systems necessitate constant supervision. Upgrading the algorithms from visual augmentation and post-operative prognosis to completely operating software is not something to be taken lightly. However, if we manage to succeed, would you be receptive to a fully autonomous robot as your surgeon? Significant barriers concerning bioethics emerge; the potential for misuse, risk assessment, supervision, referrals, the need to respect and protect patient autonomy and transparency while using the algorithms, and above all the understanding of the dynamics of illness and the human condition. So, can we provide a simple response to such a prime issue? The truth is, we cannot provide an answer for the future where an answer cannot be delivered effortlessly.

## Editorial

From the very beginning of human existence, mankind always wanted to employ semi- or even fully-automated apparatuses in an attempt to “tame” life itself [1]. Since then, much progress has been made; computers have been invented, large data sets are now manipulated at a glance, complex human-simulating models are made, artificial intelligence (AI) and machine learning have come to the frontline, paving the way toward a future of healthcare and operational efficiency [2]. These “tools” with their ability to process large amounts of structured and unstructured data, produce meaningful outputs, which can then be utilized by physicians and computers to enhance the decision-making process, minimize inaccuracies, and provide an overall healthcare service of high quality [3]. Outputs, which are fundamental for robotic surgery as they “teach” the system to accomplish the desirable outcome of interest, while reducing surgical mishaps [3], and to resemble a human brain; a much faster one in terms of the multiple connection levels [4]. 

The substantial contribution of AI in the field of surgery could be simplified into five labels: amelioration of the pre-operative assessment, evaluation of the surgical risk, post-operative outcome prediction, optimization of the intraoperative procedure, and boost to research [5]. Under this scenery and aligning with the current flow, cardiac surgery is constantly evolving [5]. While there has been immense progress in almost every one of the aforementioned topics, and while surgical robotic platforms, such as the da Vinci system, are already implemented in various cardiac procedures, for example, robot-assisted coronary, mitral, or aortic valve surgery [1], a question remains [5]. Will there be a time when robots will replace cardiac surgeons? Despite the complexity of the subject, the answer seems rather straightforward. Current evidence tends to support the opposite [4,5], but as we are still learning from AI, this might change in the future. So, first, let us take a step back.

The currently used operating systems may have successfully managed to reduce the intraoperative length and to increase precision and efficacy, but necessitate constant supervision [5]. Upgrading the algorithms from visual augmentation and post-operative prognosis to completely operating software [1] is not something to be taken lightly. These machines must learn not only the intricate anatomy of the human body, the stages of the procedure and to imitate the delicate actions of the surgeon´s hands [1], but must apply all their input in the complex, clock-ticking, surgical field. Under any circumstances, for example, whether there is active bleeding, or a not-so-convenient visual angle [2], they must succeed. Getting a robot to execute simple surgical maneuvers and tasks like suturing is demanding, yet feasible. Getting a robot to perform an entire surgery, substituting the surgical team would be herculean and beyond reach [5].

In addition, AI tools are far too long from a universal implementation [3-5]. Notwithstanding the economic burden of the installation, operation, and maintenance of such technologies, one should keep in mind that all these outputs must first be thoroughly tested and validated [4]. Their successful application reflects on the level of the provided healthcare services and most importantly depends on the ability of the physicians to interpret and employ the available results [4]. To make a good approximation of their effectiveness, numerous statistical models and tests are available in the armamentarium of the machine learning technicians, including but not limited to the “area under the receiver operator curve” and the “area under the precision-recall curve” [5]. Nonetheless, a unanimous “validating” consensus is yet to be reached [3]. For the second part, adequately trained doctors and in our case heart surgeons are the key element [1]. From both sides of the Atlantic Ocean, the societies of cardio-thoracic surgeons have initiated “subspecialty training and fellow programs,” providing surgeons with valuable expertise, in order to establish successful robotic programs [1]. However, such programs are still limited or/and of very short duration.

Would you be receptive to a robot being autonomously operating on you, if we somehow manage to overcome all the above "obstacles"? Let us have a look in the operating room.

The energy flows, the lights illuminate, the cameras depict, the tools are prepared, the monitors display the environment and the AI seems ready to decide about actions and participate or even conduct the operation. Meanwhile, the senses form the perception, the eyes behold, the hands press and the brain calculates for everything to be in order. Shall medicine let robots and AI alone in the constructed and organized bio-chaos of the human organism [6]? Shall humans allow machines controlled by evolved software to manage our species' health? This shift in medicine produces maps of trust towards AI, a cluster of predictors of trust and theories which manifest individual antecedents such as AI reliability and anthropomorphism. Algorithms controlling summated information inputs are here to take their place pacing in our footsteps with a goal to be our footsteps, an embodied AI [6]. Significant barriers concerning bioethics violently emerge in front of us, the potential for misuse, risk assessment, supervision, referrals, and the need to respect and protect patient autonomy, transparency in the use of algorithms, and above all the understanding of the dynamics of illness and human condition [7]. Can we provide a simple response to such a prime issue? Well, we cannot provide an answer for the future where an answer cannot be delivered effortlessly. The balance between progress and man should be gauged on a scale made of health perseverance and bioethical conservation.
